# Rate my Editor

**DOI:** 10.1101/gad.350445.123

**Published:** 2023-01-01

**Authors:** Titia de Lange

**Affiliations:** Rockefeller University, New York, New York 10021, USA

It is difficult to imagine *Genes & Development* without Terri Grodzicker and vice versa. Terri embodied *G&D*. During Terri's reign, *G&D* became a steadfast, reliable journal that knows what it is (and what it is not). It stands for high-quality, high-interest, serious science. Terri avoided gimmicks and was not swayed by fashions. She was not tempted to start an extended family of *G&D*-adjacent journals. And, in my experience, Terri was great as an Editor (more about that below). Now that she is stepping down from her position as Editor, I have a suggestion for Terri: Please consider running ratemyeditor.org with me. Here is what I have in mind.

Once I retire, or so I have told my friends, I will set up a website where biologists can provide information regarding their publishing experiences and find out which Editors are rated highly. The domain name I registered is ratemyeditor.org. According to my plan, some day in the future, anyone will be able go to ratemyeditor.org, look up a journal, and find out which one of its Editors are appreciated by the community.

The idea is that ratemyeditor.org will accumulate knowledge crowdsourced from scientists’ comments regarding their experience with the editorial process. They would be asked for relevant metrics such as time from submission to when the manuscript went out for review (or was editorially rejected), how long it took to receive the referee's comments and decision letter, and, in case of a second round of reviews, how long that process took.

Additional questions will concern the Editor's level of engagement. Did the Editor give the impression of having understood the paper? Did the Editor select reviewers who were knowledgeable (as gleaned from their comments)? Was the Editor responsive (e.g., how long did it take to get a response to an e-mail, and was the Editor willing to discuss the reviews or the editorial rejection)? Did the Editor insist that you do all 57 experiments suggested by the referees? Was the Editor willing to overrule a reviewer who was clearly biased or uninformed? Was the Editor willing to seek an additional referee? How many reviewers were consulted? You get the idea. Before this information would become publicly available, we (Terri and I?) would verify the identity of the respondent who would, of course, have the option to remain anonymous. Only journals with professional Editors would be considered (e.g., not the *PNAS*).

**Figure:**
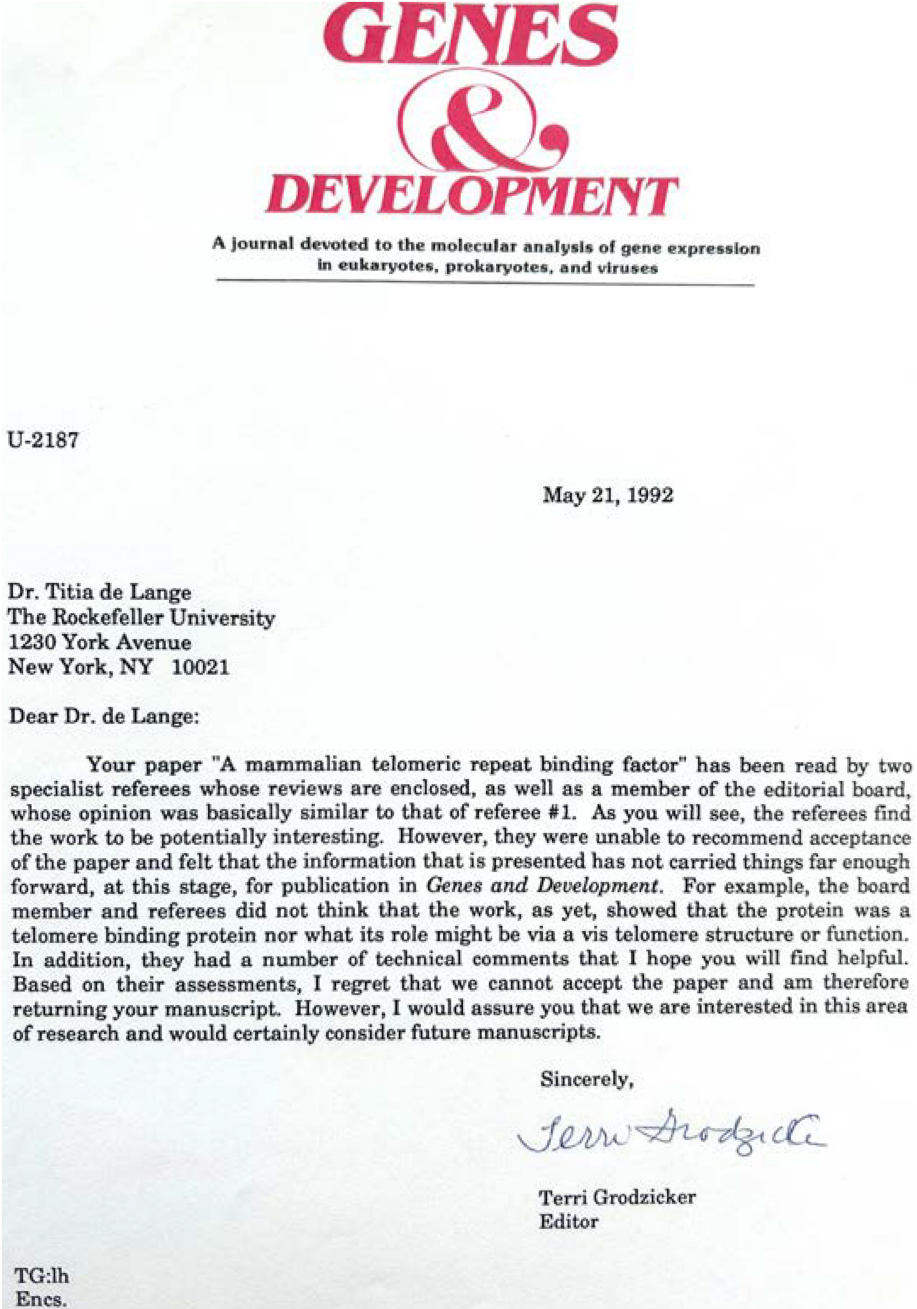
*Rejection letter of the first manuscript I submitted to* G&D. *The paper, published later that year in* MCB, *described a DNA binding factor specific for telomeric repeats. The reviewers felt that the factor should be purified, cloned, and shown to bind to telomeres. Those experiments took me another 3 years and were published in* Science. *The protein, now known as TRF1, was the first shelterin component we identified. I can't recall my feelings reading the rejection letter as a newly minted Assistant Professor at the Rockefeller University, but I doubt they included the warm feelings and respect toward Terri that I have today.*

Why do this? Because some Editors are great and some are not so great, and most of us try to avoid the latter. Terri Grodzicker was a prime example of the first category. She read your manuscript and decided quickly whether to send it out for review, often after consulting a few trusted advisors in the field. She usually only contacted two reviewers and, more often than not, they were the right people to look at the manuscript. She chased the reviewers, and their comments usually arrived within a few weeks. Her editorial decisions were guided by the referees but not determined by them. She understood the science and therefore could come to her own conclusion about the value of the work. She responded to e-mails about the manuscript almost immediately and was willing to listen to arguments regarding its merits. However, it was always clear that she was the one who made the decision on whether to publish.

Did she accept all my papers? No (as shown here ). Was she always right? Not according to Terri, who told me that she regretted rejecting one of our papers. Nobody is always right. Terri had taste, a key feature of a great Editor. She dealt with each manuscript in a straightforward and transparent way. Being embedded at Cold Spring Harbor Laboratory with its great science and its renowned meetings, she knew more scientists than most Editors and she knew life in the trenches of biological research. Perhaps because of this, she understood that some of the 57 additional experiments requested by the reviewers were merely going to torture the first author and not improve the paper. She would tell you which ones she considered critical.

Thank you, Terri. Science needs more great Editors like you. I will miss you as an Editor but hope to see you at forthcoming CSHL meetings. So, how about running ratemyeditor.org with me after I throw in the towel?

